# Protective Effects of 6-Gingerol on Cardiotoxicity Induced by Arsenic Trioxide Through AMPK/SIRT1/PGC-1α Signaling Pathway

**DOI:** 10.3389/fphar.2022.868393

**Published:** 2022-04-28

**Authors:** Xue Han, Yakun Yang, Muqing Zhang, Xi Chu, Bin Zheng, Chenxu Liu, Yucong Xue, Shengjiang Guan, Shijiang Sun, Qingzhong Jia

**Affiliations:** ^1^ School of Pharmacy, Hebei University of Chinese Medicine, Shijiazhuang, China; ^2^ College of Integrative Medicine, Hebei University of Chinese Medicine, Shijiazhuang, China; ^3^ The Fourth Hospital of Hebei Medical University, Shijiazhuang, China; ^4^ Affiliated Hospital, Hebei University of Chinese Medicine, Shijiazhuang, China; ^5^ School of Basic Medicine, Hebei University of Chinese Medicine, Shijiazhuang, China; ^6^ School of Pharmacy, Hebei Medical University, Shijiazhuang, China

**Keywords:** AMPK/SIRT1/PGC-1α pathway, arsenic trioxide, cardiotoxicity, 6-gingerol, oxidative stress

## Abstract

**Background and Objective:** Arsenic trioxide (As_2_O_3_) induced cardiotoxicity to limit the clinical applications of the effective anticancer agent. 6-Gingerol (6G) is the main active ingredient of ginger, a food with many health benefits. The present study aims to investigate the potential pharmacological mechanisms of 6G on As_2_O_3_-induced myocardial injury.

**Methods and Results:** Fifty KunMing mice were divided into five groups (*n* = 10) receiving: 1) physiological saline; 2) 6G (20 mg/kg) alone; 3) As_2_O_3_ (5 mg/kg); 4) 6G (10 mg/kg) and As_2_O_3_ (5 mg/kg); 5) 6G (20 mg/kg) and As_2_O_3_ (5 mg/kg). 6G was given orally and As_2_O_3_ was given intraperitoneally once per day for seven consecutive days. Biochemical, histopathological, transmission electron microscopy, ELISA, and western blotting analyses were then performed. Based on the resultant data, As_2_O_3_ was found to induce cardiotoxicity in mice. 6G significantly ameliorated As_2_O_3_-induced heart injury, histopathological changes, oxidative stress, myocardial mitochondrial damage, inflammation, and cardiomyocyte apoptosis, while reversed As_2_O_3_-induced inhibition of the AMPK/SIRT1/PGC-1α pathway.

**Conclusion:** Our experimental results reveal that 6G effectively counteracts As_2_O_3_-induced cardiotoxicity including oxidative stress, inflammation and apoptosis, which might be attributed to its activation action on AMPK/SIRT1/PGC-1α signaling pathway.

## Introduction

As_2_O_3_ is a traditional Chinese medicine that has been used in China for more than 2,400 years. In recent years, reports have emerged that As_2_O_3_ may be effective at treating a wide array of cancers, and it has been used as a more suitable drug for the treatment of acute promyelocytic leukemia (Wang X. et al., 2021; Zong et al., 2021). However, As_2_O_3_ can cause various diseases such as cardiovascular disorder, and As_2_O_3_ toxicity presented significant obstacles to its clinical applications (Emadi and Gore, 2010). It has been reported that As_2_O_3_-induced cardiotoxicity is regulated by reactive oxygen species (ROS) (Ficker et al., 2004; Ghosh et al., 2009), which in turn leads to pro-/anti-oxidant imbalances, such as increased malondialdehyde (MDA) and decreased superoxide dismutase (SOD), glutathione (GSH) and catalase (CAT). Elevation of MDA is a very useful indicator of oxidative stress (C. Zhang et al., 2021). SOD, GSH and CAT are all important free radical scavengers that exert antioxidant functions and protect cells from damage (Birari et al., 2020). However, there is no definitive clinical drug to treat As_2_O_3_-induced cardiotoxicity.

As a highly conserved serine/threonine-protein kinase, AMP-activated protein kinase (AMPK) has a triggered effect on the metabolism of bioenergy (Salt and Hardie, 2017). When AMPK is activated, it could monitor the function of mitochondria and the energy status of the cell (Bechard et al., 2010) and regulate the activity of the silent information regulator 1 (SIRT1). Then the peroxisome proliferator-activated receptor-gamma coactivator one alpha (PGC-1α) is activated after activation of AMPK and SIRT1. The AMPK/SIRT1/PGC-1α pathway plays an important role in oxidative stress, and mitochondrial biosynthesis (Canto and Auwerx, 2009; Birari et al., 2020).

**FIGURE 1:**
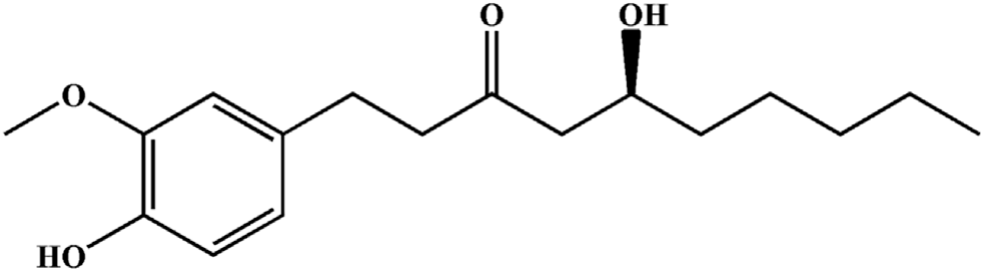
Chemical structure of 6G.

Ginger is a widely used spice that exhibits several health benefits. It is used as a home remedy in the treatment of stomach disorders with significant value (Haniadka et al., 2013; Seif et al., 2021). The gingerol-like compounds are the main bioactive substances in the non-volatile, pungent components of ginger. 6-Gingerol (6G) is one of the major components of total gingerols extracted from ginger (Luo et al., 2021), and its chemical structure of 6G is shown in Figure 1. Specifically, 6G has been shown to be potentially efficacious against cancer (Kapoor et al., 2016; Luna-Dulcey et al., 2018) and to possess anti-inflammatory and anti-apoptotic properties (Krell and Stebbing, 2012; Zhang et al., 2017). Previous studies by our group have shown the protective effect of 6G *in vitro* and *ex vivo* models, and proposed that the cardioprotective and antioxidant properties of 6G may be a result of decreased intracellular Ca^2+^ via the suppression of Ca^2+^ influx and contractility in ventricular myocytes and inhibition of the TLR4/MAPKs/NF-κB pathway (Han et al., 2019; Han et al., 2020). These studies indicate that 6G possesses cardioprotective potential, while the exact effects and potential protective mechanisms of 6G in As_2_O_3_-induced cardiotoxicity remain to be fully elucidated. Hence, the present study aimed to explore the effects and underlying pharmacological mechanisms of 6G on As_2_O_3_-induced oxidative stress, inflammation, and apoptosis of cells.

Herein, a mouse model of As_2_O_3_ induced-cardiotoxicity was established to explore whether 6G regulates oxidative stress, inflammation, and apoptosis via the AMPK/SIRT1/PGC-1α signaling pathway. To date, the role of 6G as an anti-cardiotoxic agent has not been demonstrated in As_2_O_3_-induced cardiotoxicity. Thus, our study explores the antioxidant and cardioprotective properties of 6G against As_2_O_3_-induced cardiotoxicity.

## Materials and Methods

### Animals

In the current study, fifty male KunMing mice (20–25 g) were used. Before the experimental application, mice were housed at 23–25°C in 12 h light/dark cycles (50–55% relative humidity) and provided with free access to water and food. All experimental procedures regarding animals were complied with the guidelines of animal experiments from the Ethics Committee of Hebei University of Chinese Medicine (China, DWLL2021098).

### Drugs and Chemicals

6G (purity >98%) was obtained from Alfa Biotechnology Co., Ltd. (Chengdu, China), and As_2_O_3_ (purity >98%) in the experiment was supplied by Shuanglu Pharmaceutical Co., Ltd. (Beijing, China). All other analytical grade reagents used in this study were provided by Sigma Chemical Company (MO, United States), unless otherwise specified.

### Experimental Design

**FIGURE 2:**
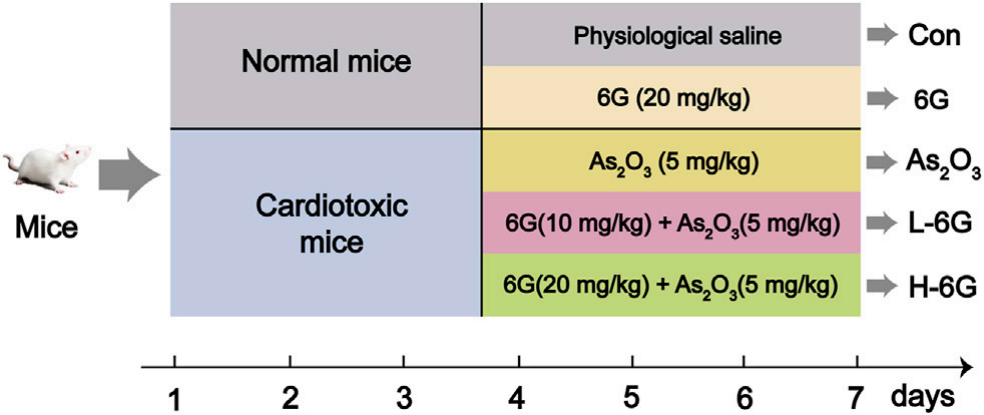
Diagram of animal experimental design.

The dose selection of 6G (El-Bakly et al., 2012; Han et al., 2020) and As_2_O_3_ (Hemmati et al., 2018; Birari et al., 2020; Jin et al., 2020) in this study was based on our preliminary experiments and previous studies as well as doses used by other research groups in animal experiments. The experimental design of all animals is depicted in Figure 2. Mice were divided into five groups, which are described below (10 mice/group):

Group 1 (Con): Injected with physiological saline as a vehicle.

Group 2 (6G): Treated with 6G (20 mg/kg) orally for 7 days.

Group 3 (As_2_O_3_): Injected intraperitoneally with As_2_O_3_ (5 mg/kg) for 7 days.

Group 4 (L-6G): Daily treated with 6G (10 mg/kg) orally, then followed by injected intraperitoneally with As_2_O_3_ (5 mg/kg) for 7 days.

Group 5 (H-6G): Daily treated with 6G (20 mg/kg) orally, then followed by injected intraperitoneally with As_2_O_3_ (5 mg/kg) for 7 days.

### Electrocardiography and Cardiac Weight Index Assessment

Briefly, mice were anesthetized with sodium urethane (1 g/kg). Electrocardiography was recorded using an electrocardiography recorder (Biological Signal Collection System, Chengdu Instrument Factory, China). Blood was then collected and subsequently the heart was removed, weighed and photographed. The heart weight-to-body weight ratio (cardiac weight index, CWI) was calculated.

### Detection of Serum Biochemical Parameters

After electrocardiography, blood samples were centrifugated at 1,500 rpm for 5 min. The serum levels of lactate dehydrogenase (LDH), creatine kinase (CK), creatine kinase-MB (CK-MB) and cardiac troponin I (CTnI) were detected with an automatic biochemical analyzer (Shenzhen Kubel Biotechnology Co., LTD., China).

### Evaluation of Histopathology

For examination, the hearts of mice were fixed in paraformaldehyde solution (4%), embedded in paraffin, cut into sections (5-μm-thick) by microtome, and stained with hematoxylin-eosin. The morphology of heart tissue was observed under ×20, ×200 and ×400 magnification with a light microscope (Nikon, Japan).

### Assessments of ROS and Antioxidant Enzymes

A fluorescent probe dihydroethidium (DHE) (Servicebio, Wuhan, China) was used to detect the production of ROS in myocardial tissue. DHE staining solution was added into frozen sections and the slices were incubated at 37°C for 30 min. The treated sections were then washed with PBS three times for 5 min each and incubated with DAPI solution in a dark environment for 10 min. Finally, the sections were observed using a fluorescence microscope (Eclipse C1, Nikon, Tokyo, Japan).

The heart tissue samples were homogenized into a 10% (W/V) homogenate and obtained by centrifugation at 3,500 rpm for 10 min. Biochemical analyses were performed using the supernatant. Levels of SOD (A001-1), MDA (A003-1), GSH (A006-2) and CAT (A007-2) were obtained by commercially available kits (JianCheng, Nanjing, China), respectively, on the basis of the manufacturer’s instructions.

### Transmission Electron Microscope

The fresh heart samples were fixed in glutaraldehyde (4%) for 2 h and rinsed with phosphate buffer (0.1 M, pH 7.4). Briefly, after being washed with phosphate buffer three times, the myocardial tissues were post-fixed in osmium tetroxide (1%, 0.1 M) for 2 h, dehydrated, and embedded in Epon 812 (TAAB). After fixation, heart samples were stained with uranyl acetate and lead citrate and visualized with an electron microscope (HT7700, Hitachi, Japan).

### Estimation of Inflammatory Cytokine Levels

ELISA testing was used to estimate inflammatory cytokine levels, including tumor necrosis factor-a (TNF-α) and interleukin-6 (IL-6). The samples of the heart were immediately homogenized in a tissue lysing device (Servicebio, Wuhan, China) and centrifuged at 3,000 rpm for 10 min at 4°C. The upper supernatant was used for all ELISA kit analyses. TNF-α (88-7324), and IL-6 (88-7064) contents were obtained from Thermo Fisher.

### Western Blot Analysis of Apoptosis and AMPK/SIRT1/PGC-1α Signaling

Western blot analysis was carried out to detect the protein expressions of apoptosis-related indicators, including Bax, Bcl-2, Caspase-3, cleaved-Caspase-3, and members of the AMPK/SIRT1/PGC-1α pathway, as described previously (Han et al., 2020). Briefly, total protein was extracted, separated on SDS-PAGE gels (10%, Servicebio, China), transferred onto a PVDF membrane (Servicebio, China). The membranes were blocked with 5% (W/V) skimmed milk TBST (pH 7.3) for 1 h at 37°C and incubated overnight in 4°C with the major antibody of anti-Bcl-2 (dilution: 1:100,000) (Servicebio, China), anti-Bax (dilution: 1:1,000) (Affinity, United States), anti-Caspase-3 (dilution: 1:1,000) (Servicebio, China), anti-cleaved-Caspase-3 (dilution: 1:1,000) (Servicebio, China), anti-AMPK (dilution: 1:1,000) (BIOSS, United States), anti-Sirt1 (dilution: 1:1,000) (San Ying, China), and anti- PGC-1α (dilution: 1:1,000) (Servicebio, China). Then the immobilized primary antibody conjugated with the secondary antibody (dilution: 1: 5,000) (Servicebio, China) in TBST for 30 min at room temperature. After a thorough washing with TBST, proteins were visualized with ECL (Servicebio, China). Quantification of protein expression was performed using the analysis software of Alpha.

### Statistical Analysis

Data are shown as mean ± SEM and analyzed via one-way analysis of variance (ANOVA), followed by Tukey’s *post hoc* test using Origin Pro version 9.1 software. Statistical significance was accepted at a value of *p* < 0.05.

## Results

### 6G Ameliorated As_2_O_3_-Induced Heart Injury

**FIGURE 3:**
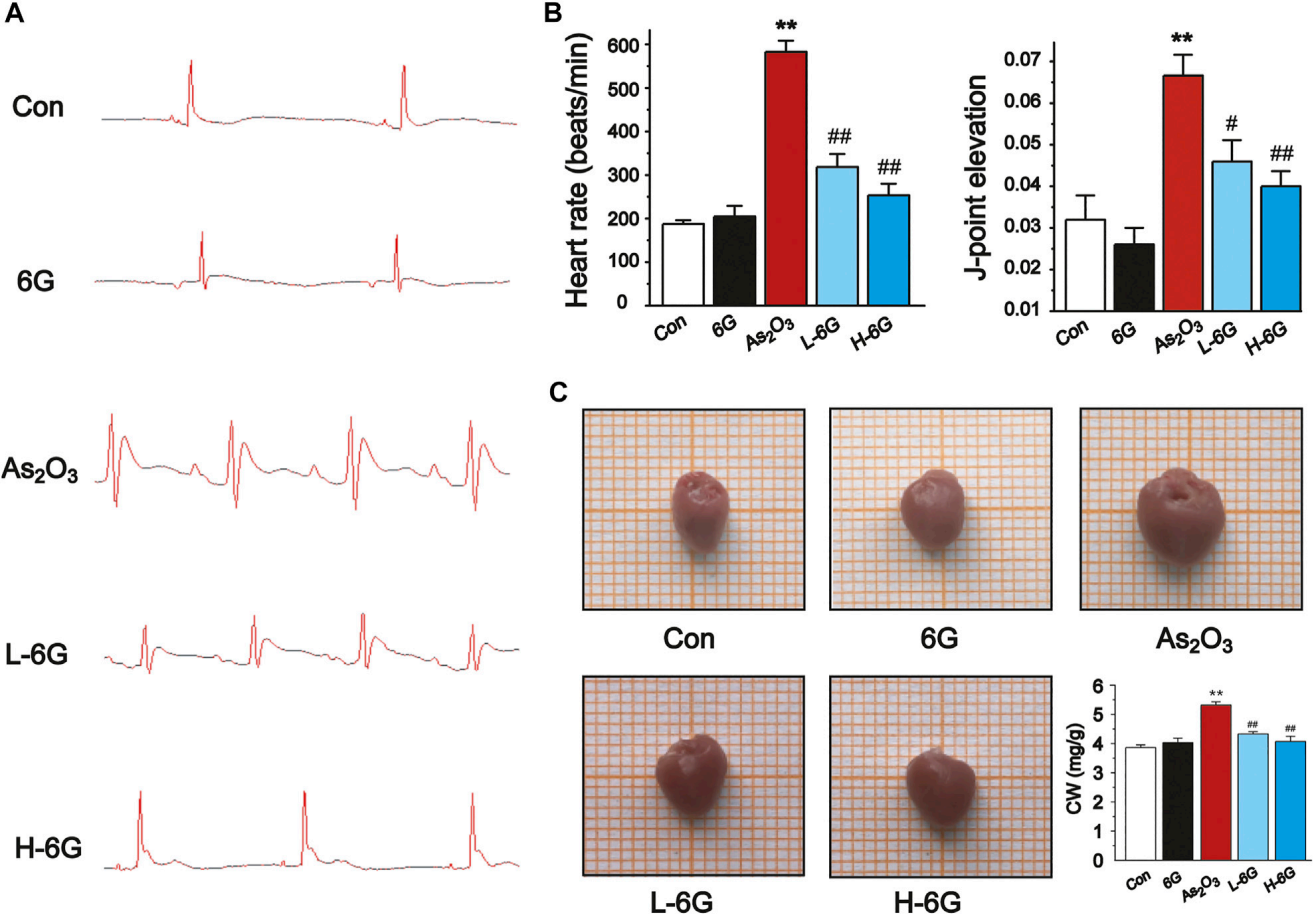
Effect of 6G on ECG, heart rate, J-point elevation, and general appearance and coefficients. **(A)** Representative ECG waveforms were presented in each group. **(B)** 6G leads to a decrease in As_2_O_3_-induced heart rate acceleration and J-point elevation. **(C)** Representative pictures of the heart appearance and statistical analysis of CW were presented in each group. Data are represented as mean ± SEM, *n* = 4–6. ^**^ *p* < 0.01 as in comparison to the Con group and ^#^ *p* < 0.05, ^##^ *p* < 0.01 as in comparison to the As_2_O_3_ group.

The ECG, body mass, and heart index of mice are shown in Figure 3. Sample tracings of ECG from the experimental animals are shown in Figure 3A. Compared to the Con group, As_2_O_3_ induced a significant rise in heart rate and ST height (Figure 3B, *p* < 0.01). L-6G and H-6G induced a prominent decrease in both heart rate and ST height (Figure 3B, *p* < 0.05 or *p* < 0.01).

As_2_O_3_ administration induced cardiac enlargement, which presented as a brownish and swollen heart. However, L-6G and H-6G significantly improved this pathological condition, with an appearance similar to that of the Con group (Figure 3C). The CWI in the As_2_O_3_-treated mice was higher than in the Con group. In contrast, 6G treatment significantly reduced the level of CWI induced by As_2_O_3_ (Figure 3C, *p* < 0.01).

**FIGURE 4:**
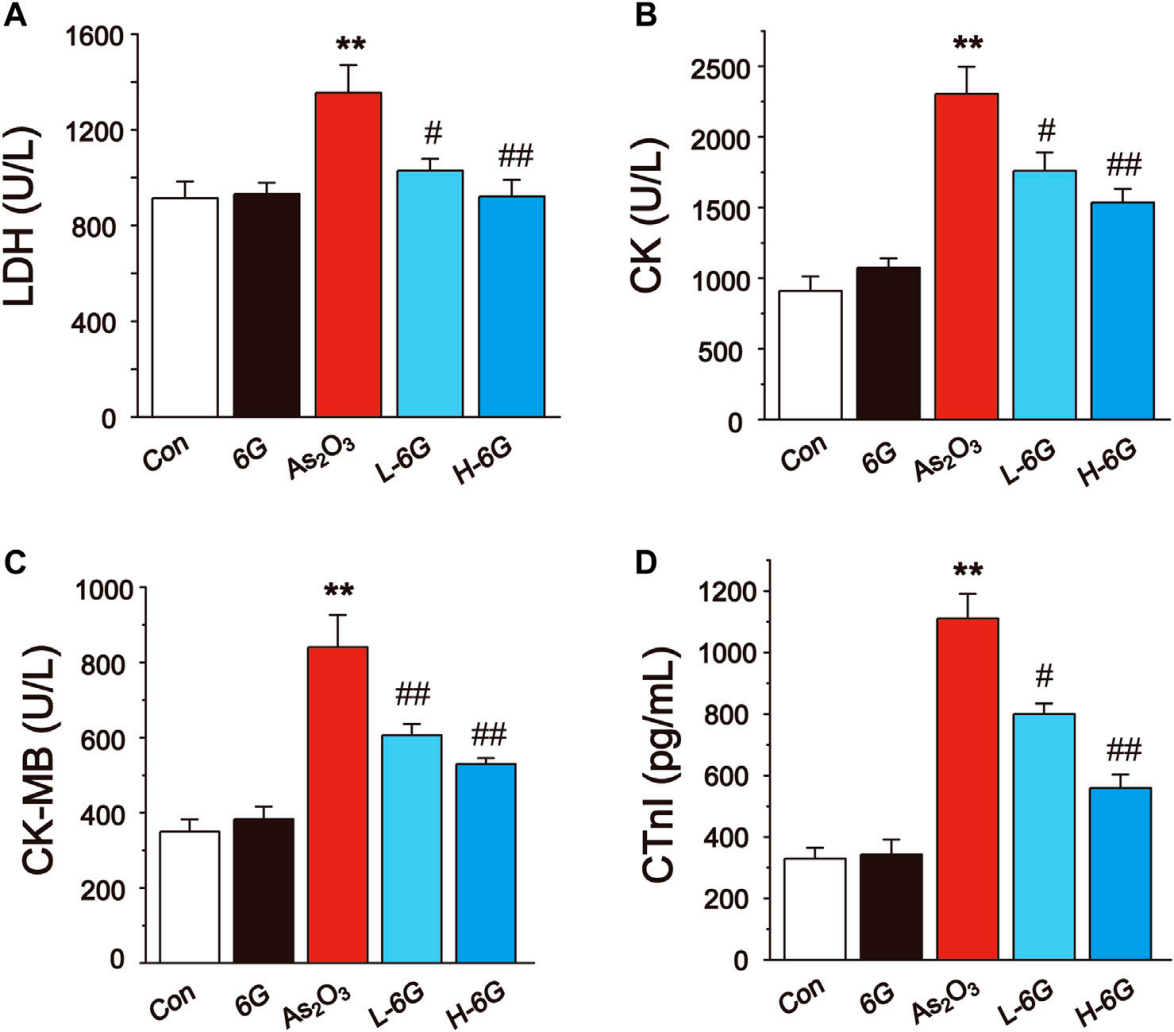
Effect of 6G on the perturbed cardiac injury markers in As_2_O_3_-treated mice. The effects of 6G on serum concentration of LDH **(A)**, CK **(B)**, CK-MB **(C)** and CTnI **(D)** in each group. Data are expressed as mean ± SEM, *n* = 6. ^*^ *p* < 0.05, ^**^ *p* < 0.01 as in comparison to the Con group and ^#^ *p* < 0.05, ^##^ *p* < 0.01 as in comparison to the As_2_O_3_ group.

The results showed that administration of As_2_O_3_ induced a significant rise in the LDH, CK, CK-MB and CTnI levels relative to Con group (Figure 4, *p* < 0.01). On the other hand, treatment with L-6G and H-6G significantly decreased the levels of these cardiac markers relative to As_2_O_3_-treated mice (*p* < 0.05 or *p* < 0.01).

### 6G Improved As_2_O_3_-Induced Histopathological Changes

**FIGURE 5:**
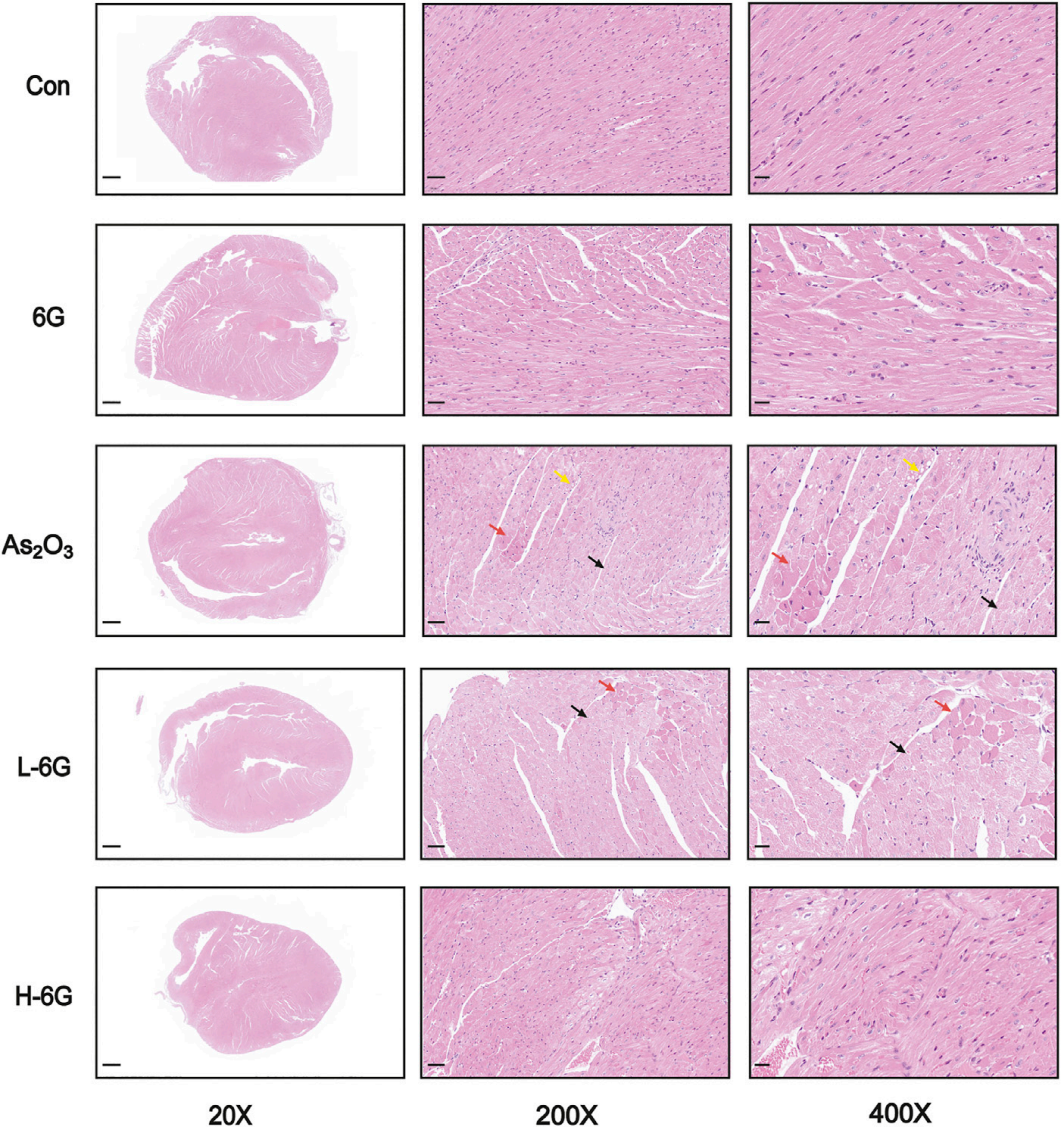
Histological examination in each group. Red arrows represent cell necrosis, yellow arrows represent edema, and black arrows represent muscle fiber loss. *n* = 6.

H&E staining, as shown in Figure 5, revealed typical myofibrillar structures in the Con group, while the myocardium of As_2_O_3_-treated mice suffered from inflammatory cell infiltration, edema, and cell necrosis. On the contrary, mice in the L-6G and H-6G groups partially alleviated the above pathological changes. Furthermore, the myocardial histoarchitecture of 6G-treated mice was similar to that of mice in the Con group.

### 6G Suppressed As_2_O_3_-Induced Oxidative Stress

**FIGURE 6:**
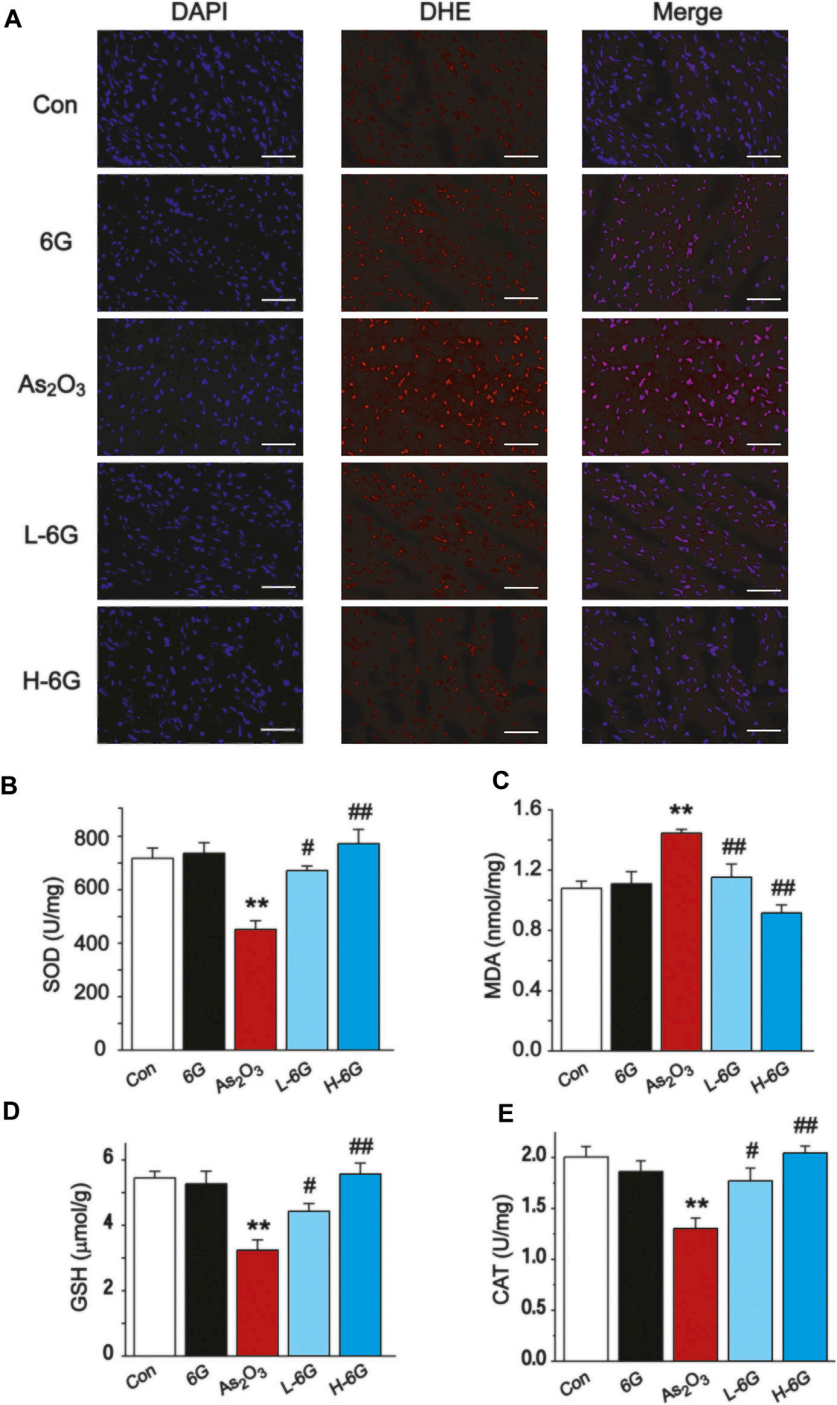
Effect of 6G on oxidative stress in cardiac tissues. **(A)** Representative ROS fluorescence images in each group (400 ×, scale bar = 50 µm). The effects of 6G on the levels of SOD **(B)**, MDA **(C)**, GSH **(D)** and CAT **(E)** in myocardial tissues of each group. Data are presented as mean ± SEM, *n* = 3. ^**^ *p* < 0.01 as in comparison to the Con group and ^#^ *p* < 0.05, ^##^ *p* < 0.05 as in comparison to the As_2_O_3_ group.

As shown in Figure 6A, our data revealed that ROS levels in As_2_O_3_-treated mice were dramatically increased in comparison to the Con group. By contrast, ROS levels decreased in the L-6G and H-6G groups in comparison to the As_2_O_3_-treated mice.

The levels of oxidative stress in heart tissue homogenates were analyzed, and it was found that SOD, GSH and CAT levels decreased in As_2_O_3_-treated mice relative to the Con group, while MDA levels increased significantly (*p* < 0.01). Conversely, 6G treatment statistically increased the levels of SOD, GSH and CAT, as well as decreased the levels of MDA compared to the As_2_O_3_-treated mice (Figures 6B–E, *p* < 0.05 or *p* < 0.01).

### 6G Attenuated Myocardial Mitochondrial Damage

**FIGURE 7:**
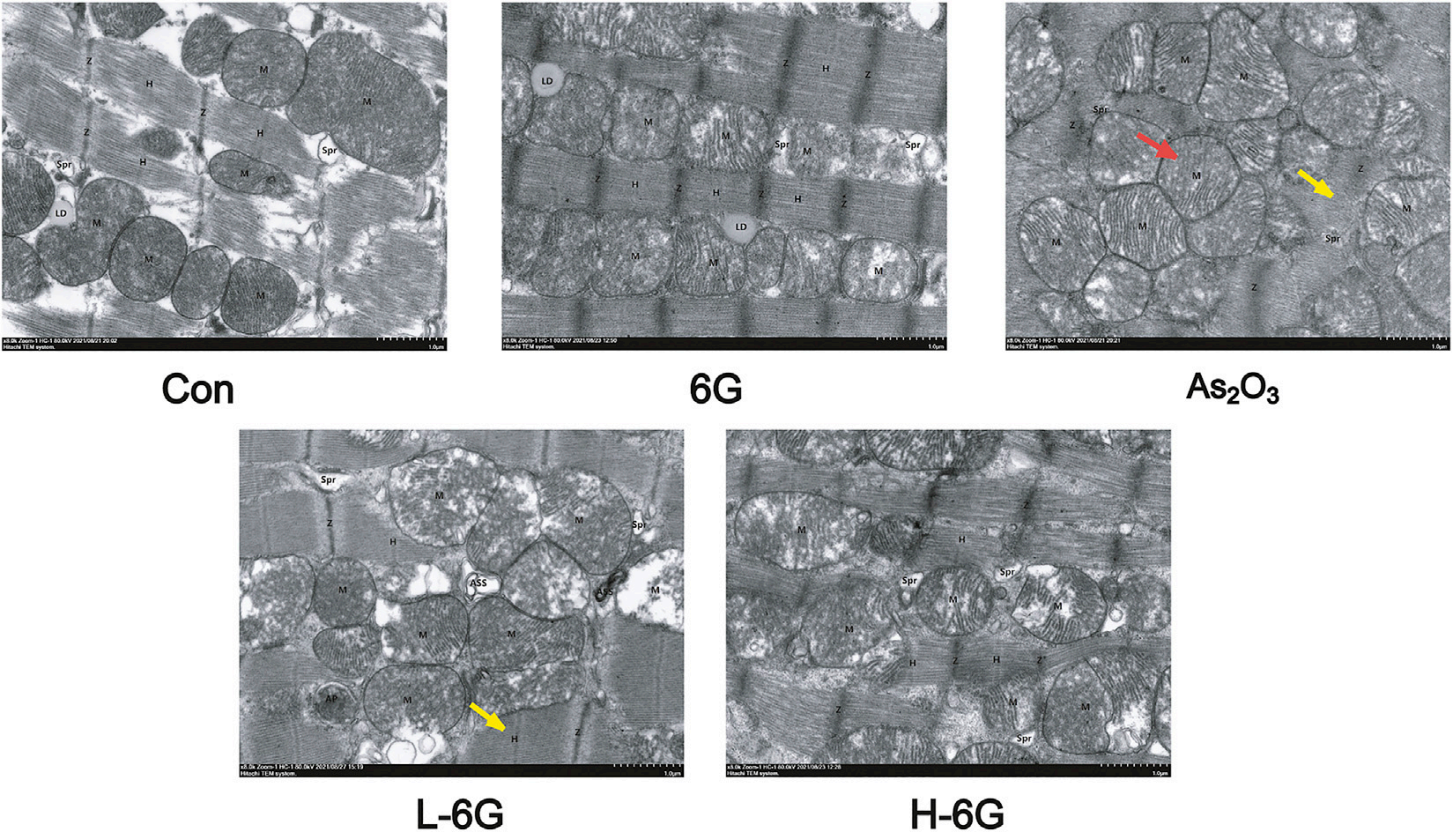
Effect of 6G on the ultrastructure of cardiomyocytes. Representative TEM images show mitochondrial injury. Red arrows represent swelling of mitochondria and yellow arrows represent rupture of mitochondrial membranes. Scale bar = 500 nm.

To investigate the protective effects of 6G on the ultrastructure of cardiomyocytes, transmission electron microscope was used to estimate the As_2_O_3_-induced cardiotoxicity. The data revealed no obvious morphological abnormalities in the Con and 6G groups. On the other hand, mitochondrial swelling and rupture of the mitochondrial membrane were found in As_2_O_3_-treated mice. However, treatment with L-6G and H-6G significantly alleviated pathological changes such as swelling of mitochondria and rupture of mitochondrial membranes (Figure 7).

### 6G Exerted Anti-Inflammatory Effects

**FIGURE 8:**
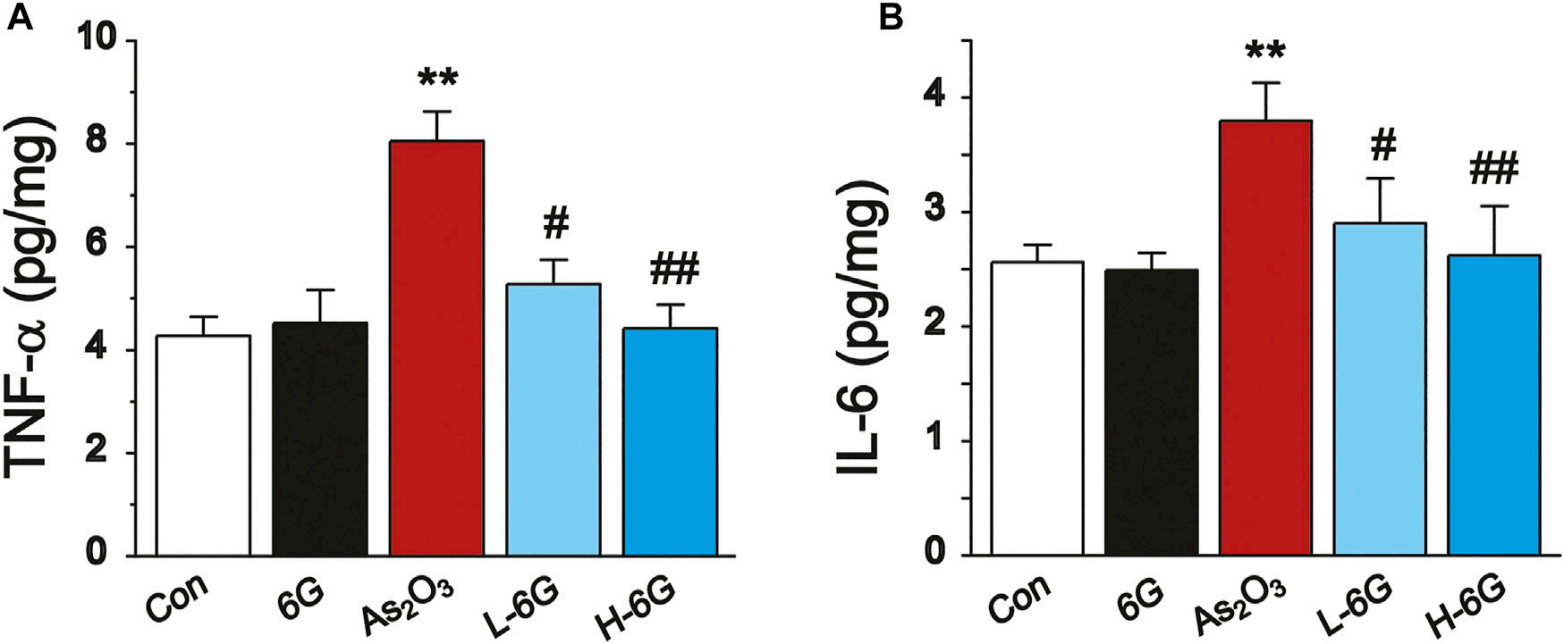
ELISA analysis of inflammatory cytokines. The effects of 6G on the levels of TNF-α **(A)** and IL-6 **(B)** in myocardial tissues of each group. Data are expressed as mean ± SEM, *n* = 3. ^**^ *p* < 0.01 as in comparison to the Con group and ^#^ *p* < 0.05, ^##^ *p* < 0.05 as in comparison to the As_2_O_3_ group.

ELISA analyses were performed to detect the inflammatory cytokines TNF-α and IL-6 in heart tissue homogenates. The levels of TNF-α and IL-6 in As_2_O_3_-treated mice were significantly higher compared with mice in the Con group (*p* < 0.01). The levels of TNF-α and IL-6 in the 6G group were similar to those of the Con group. Treatment of L-6G and H-6G suppressed the As_2_O_3_-induced rise in levels of TNF-α and IL-6 (*p* < 0.05 or *p* < 0.01). The expression levels of these cytokines among the experimental groups are shown in Figure 8.

### 6G Inhibited As_2_O_3_-Induced Heart Apoptosis

**FIGURE 9:**
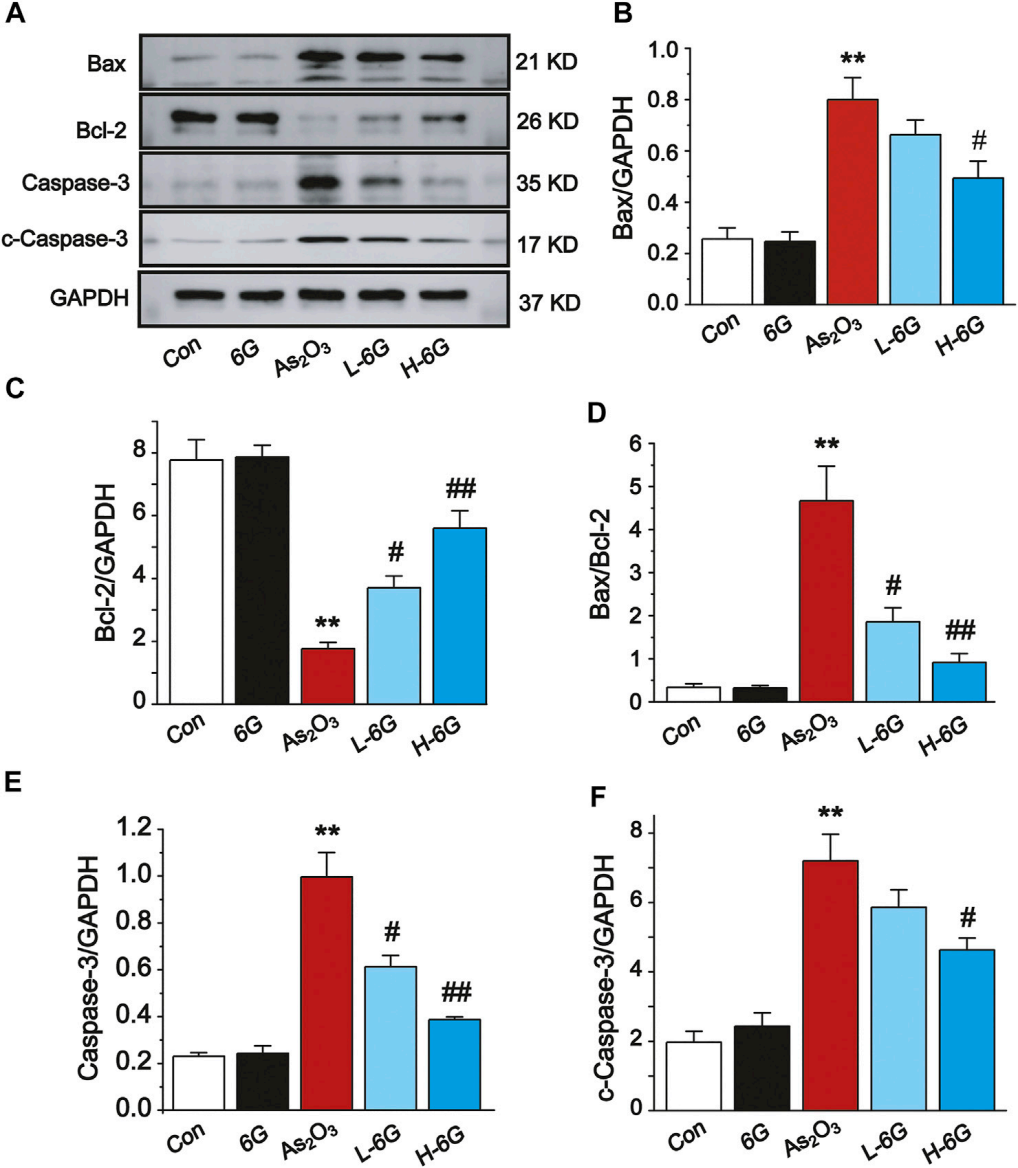
Western blot analysis of Bcl-2, Bax, Caspase-3, and cleaved-Caspase-3 in cardiac homogenates prepared from mice, **(A)** The typical protein expression bands of each group. Bar graph of the expressions of Bax **(B)**, Bcl-2 **(C)**, Bax/Bcl-2 **(D)**, Caspase-3 **(E)** and c-Caspase-3 **(F)** in myocardial tissues. Data are expressed as mean ± SEM, *n* = 3. ^**^ *p* < 0.01 as in comparison to the Con group and ^#^ *p* < 0.05, ^##^ *p* < 0.05 as in comparison to the As_2_O_3_ group.

Next, the expression levels of apoptosis-related markers were assessed to further elucidate the role of 6G in As_2_O_3_-induced cardiac dysfunction. As shown in Figure 9, Western blot analysis of apoptosis-related regulatory proteins revealed that the protein expression levels of Bax, Caspase-3 and cleaved-Caspase-3 and the ratio of Bax/Bcl-2 were obviously up-regulated, whereas the expression of Bcl-2 was down-regulated in As_2_O_3_-treated mice compared with mice in the Con group (*p* < 0.01). On the other hand, treatment with L-6G and H-6G significantly downregulated apoptosis-related regulatory proteins (i.e., Bax, Bax/Bcl-2, Caspase-3, and cleaved-Caspase-3) and upregulated the level of Bcl-2 compared with As_2_O_3_-treated mice (Figure 9, *p* < 0.05 or *p* < 0.01). Our findings indicated no significant change in mice treated with 6G alone relative to mice in the Con group.

### 6G Reversed As_2_O_3_-Induced Inhibition of the AMPK/SIRT1/PGC-1α Pathway

**FIGURE 10:**
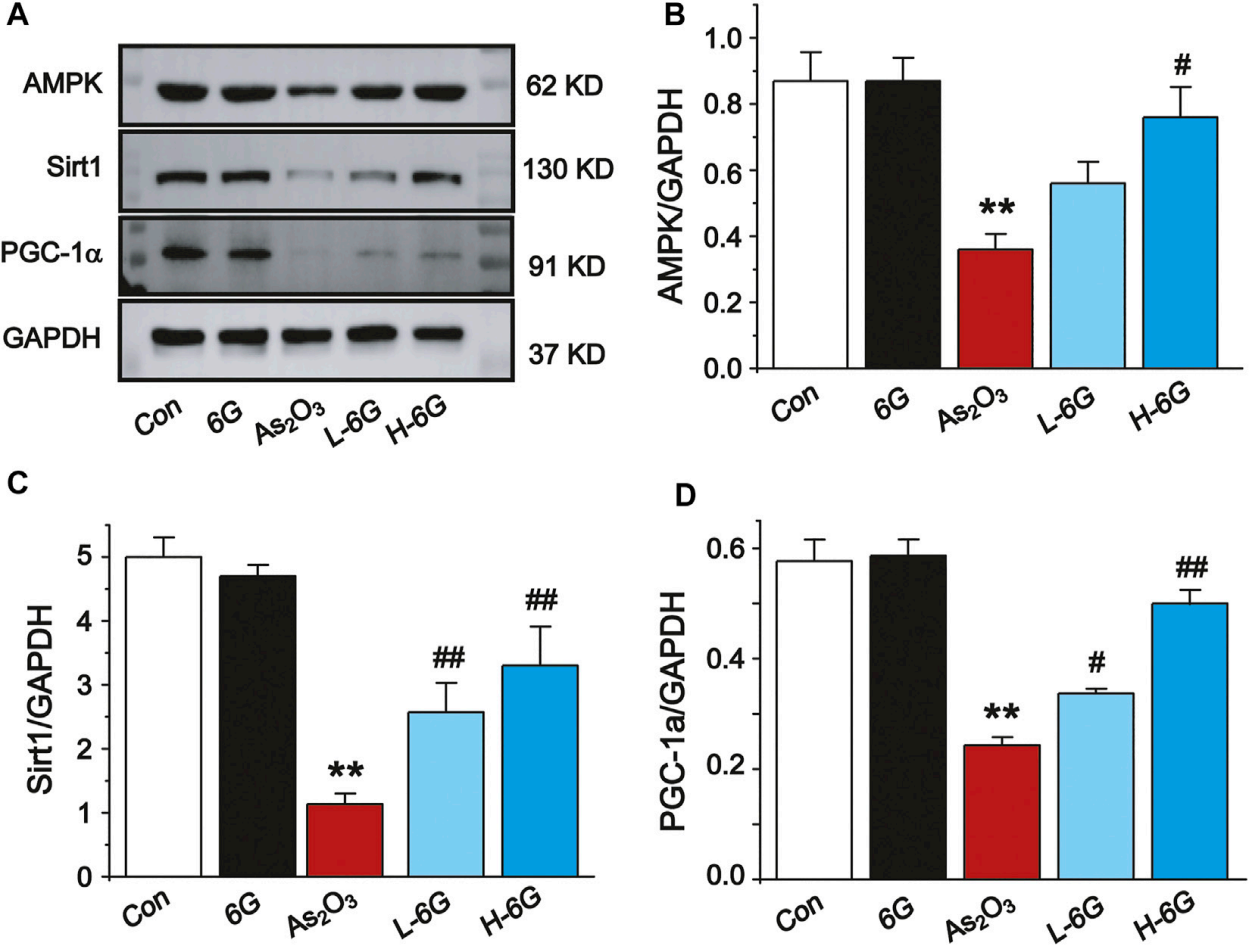
Western blot analysis of AMPK, SIRT1, and PGC-1α in cardiac homogenates prepared from mice, **(A)** The typical protein expression bands of each group. Bar graph of the expressions of AMPK **(B)**, Sirt1 **(C)** and PGC-1α **(D)** in myocardial tissues. Data are expressed as mean ± SEM, *n* = 3. ^**^ *p* < 0.01 as in comparison to the Con group and ^#^ *p* < 0.05, ^##^ *p* < 0.05 as in comparison to the As_2_O_3_ group.

Finally, the expressions of AMPK, SIRT1 and PGC-1α in each group was examined by Western blot analysis to investigate the potential mechanism of action of 6G to alleviate As_2_O_3_-induced cardiotoxicity. Further analysis indicated that As_2_O_3_ significantly downregulated the expressions of AMPK, SIRT1, and PGC-1α, while treatment with L-6G and H-6G showed an inverse trend (Figure 10). Our data indicate that 6G can suppress As_2_O_3_-induced cardiotoxicity might be through activating of the AMPK/SIRT1/PGC-1α pathway.

## Discussion

As_2_O_3_, used in ancient Chinese medicine for more than 2,000 years, plays a vital role in the treatment of various diseases such as cancer (Hoonjan et al., 2018). Ginger is one of the oldest spices and is also used as a traditional Chinese medicine due to its many health benefits. The 6G extracted from ginger is the most pharmacologically active compound in gingerol (Luettig et al., 2016; de Lima et al., 2018). Our previous experimental findings have shown that 6G can effectively inhibit inflammation and apoptosis in cardiac fibrosis and myocardial injury (El-Bakly et al., 2012; Han et al., 2020). Furthermore, 6G was shown to be capable of reversing impaired insulin signaling in arsenic-intoxicated mice (Chakraborty et al., 2012). Moreover, consistent with previous research, our study found that 6G treatment can alleviate As_2_O_3_-induced heart injury in mice by reducing oxidative stress, the inflammatory response, and apoptosis. In addition, mice treated with 6G obtained these effects via activation of the AMPK/SIRT1/PGC-1α pathway.

The current study may help guide future research on 6G or suitable derivatives for the effective prevention of As_2_O_3_-induced cardiotoxicity. Evidence has amassed that heart rate and ST height are frequently used as important indicators for normal cardiac functioning and that elevated heart rate and ST segment changes indicate damage of the myocardium (Zenger et al., 2021). As_2_O_3_-induced myocardial injury is manifested by increased heart rate and ST-segment elevation (Sonawane et al., 2018). Our results showed that As_2_O_3_ treatment significantly increased heart rate and ST height, however, 6G reversed these pathological changes, suggesting that 6G protects myocardium from As_2_O_3_-induced injury (Figure 3). Meanwhile, the 6G-alone administration group was designed to observe changes in mice before and after 6G treatment. Our results showed no significant changes in mice treated with 6G alone compared to the Con group, indicating that 6G is a safe therapeutic agent.

The utilization of As_2_O_3_ in cancer patients causes various cardiotoxic effects (Yu et al., 2017). Serum levels of LDH and CK are indicators of myocardial damage and are frequently used to estimate cardiotoxicity (Saad et al., 2020). During cardiovascular disease or injury, the levels of CK-MB and CTnI in cardiac tissues are dramatically increased (Wang R. et al., 2021). We preliminarily analyzed the toxic effects of arsenic trioxide on the heart by the level of cardiac diagnostic markers. The data from our study demonstrate that As_2_O_3_ exposure increased serum levels of LDH, CK, CK-MB and CTnI. Elevated levels of these markers indicate cardiac damage, however, treatment with 6G can significantly reduce the levels of these indicators (Figure 4). We further observed the histopathological damage caused by As_2_O_3_. Results in the present study indicated that L-6G and H-6G treatment had curative effects on histopathological changes (Figure 5). Consistent with previous research, the protective effect of 6G, as determined by heart function markers, has been reported against ISO-induced cardiotoxicity (Han et al., 2020). 6G ((S)-5-hydroxy-1-(4-hydroxy-3-methoxyphenol)-3-decanone) extracted from ginger is the most pharmacologically active compound in gingerol, and the active part of the molecule is an aliphatic chain molecule containing a hydroxyl group (Yang et al., 2010; Wang et al., 2014). Due to its cardioprotective effect, 6G can be considered a promising molecule for the treatment of drug and chemical-induced cardiotoxicity and/or other cardiovascular diseases.

Production of reactive oxygen species, depletion of antioxidants, impairment of mitochondrial function, and induction of apoptosis are involved in the pathogenesis of cardiotoxicity caused by As_2_O_3_. Mitochondria are the major site of ROS production (Bugger and Pfeil, 2020), and exposure of As_2_O_3_ is able to induce overproduction of intracellular ROS, which triggers oxidative stress-related cascades, inflammatory responses, apoptosis, and cellular membrane damage (Zhong et al., 2021). The high levels of intracellular ROS in As_2_O_3_-treated mice were associated with the impaired mitochondrial reduction of molecular oxygen (Ahangarpour et al., 2017). The levels of SOD, CAT and GSH in the tissues indicated the extent of antioxidant defense. The results of the present study also demonstrate that As_2_O_3_ can generate large amounts of ROS, which leads to a significant decrease in the levels of antioxidants (SOD, GSH and CAT), as well as an increase in the levels of lipid peroxides MDA. This suggests that As_2_O_3_ induces cardiac injury by inducing oxygen radical production and reducing endogenous protective antioxidant capacity (Figure 6). Mitochondrial dysfunction is the main cause of As_2_O_3_-induced damage. 6G treatment could reduce the swelling of mitochondria and the breakage of mitochondrial membrane (Figure 7). Our results suggest that 6G could maintain mitochondrial function.

Excessive production of ROS and induction of oxidative stress-related signaling cascades result in the overproduction of pro-inflammatory mediators. TNF-α and IL-6, both of which play key roles in the inflammatory process (Forrester et al., 2018). Inflammatory cytokines were found to be upregulated in As_2_O_3_-induced cardiotoxicity. Our results are in consistent with previous studies. Notably, TNF-α and IL-6 were significantly downregulated by 6G, suggesting that 6G exerts a significant anti-inflammatory effect by inhibiting the levels of TNF-α and IL-6 (Figure 8).

Oxidative stress caused by As_2_O_3_ results in dysfunction of mitochondria and apoptosis of cardiomyocytes, both of which contribute to the development of myocardial damage (Zhu and Zuo, 2013). It is well known that the apoptosis-related regulatory proteins Bax, Bcl-2, Caspase-3 and cleaved-Caspase-3 are closely associated with As_2_O_3_-induced cardiotoxicity. As a classical anti-apoptotic mediator, Bcl-2 can inhibit the activity of Caspase-3 which, in turn, inhibits cellular apoptosis. It is noteworthy that Bax upregulates the permeability of mitochondrial membranes, causing cytochrome ϲ release, which induces cellular apoptosis through activation of Caspase-3 and cleaved-Caspase-3 (Polat and Karaboga, 2019). We also found that As_2_O_3_ induced apoptotic damage to the heart, as evidenced by notable increases in Caspase-3, Bax, and Bcl-2 expression and the Bax/Bcl-2 ratio in the heart of As_2_O_3_-treated mice. However, in the groups treated with 6G, Bax, Caspase-3, and cleaved-Caspase-3 levels were downregulated and the Bax/Bcl-2 ratio decreased in heart tissue, while Bcl-2 expression in heart tissue was upregulated relative to As_2_O_3_-treated mice (Figure 9).

**FIGURE 11:**
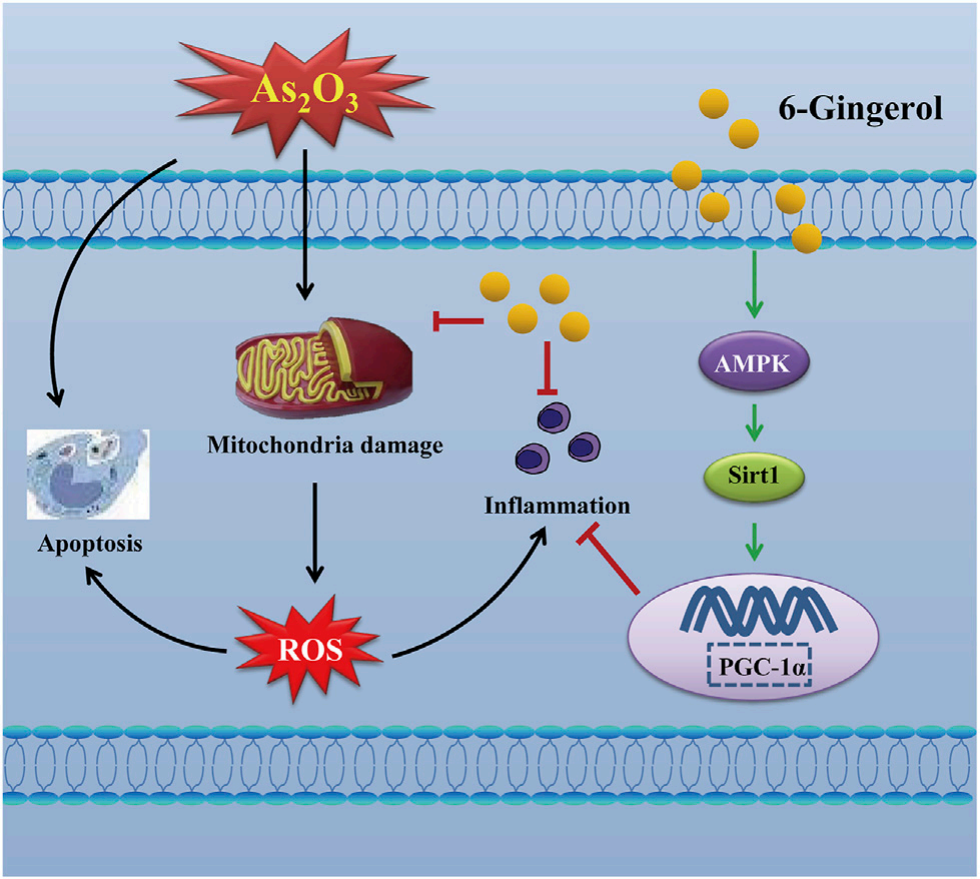
Mechanism of 6G on As_2_O_3_-induced cardiotoxicity.

The signaling cascade mediated by AMPK, SIRT1 and PGC-1α can inhibit ROS production and inflammatory cytokines (Thirupathi and de Souza, 2017). Thus, activation of the cascade may be an effective therapy against As_2_O_3_-induced damage. Multiple studies have shown that AMPK could affect the activity of the downstream molecules SIRT1 and PGC-1α, as well as the activation of AMPK and PGC-1α, which could also be regulated by SIRT1 (Tian et al., 2019). PGC-1α plays an important role in cardioprotective therapies and is highly expressed in cardiac myocytes (Lehman et al., 2000). Furthermore, PGC-1α is a key factor for myocardial mitochondrial biogenesis and for regulating the expression of the downstream proteins involved in oxidative stress (Choi et al., 2017; Di et al., 2018). SIRT1, a histone deacetylase, plays an important role in regulating a wide range of critical biological functions, including oxidative stress, energy metabolism, apoptosis, and autophagy. Our present study found that As_2_O_3_ significantly decreased the protein expressions of AMPK, SIRT1, and PGC-1α, whereas 6G upregulated the expressions of AMPK, SIRT1, and PGC-1α, enhanced SOD activities, and suppressed the production of ROS and MDA to maintain mitochondrial function and control oxidative stress (Figures 10, 11).

## Conclusion

In line with these findings, the data generated by our study suggests that 6G treatment exerts cardioprotective effects by attenuating oxidative stress, inflammation, and apoptosis. 6G may be a promising therapeutic agent for the prevention of As_2_O_3_-induced cardiotoxicity, and the AMPK/SIRT1/PGC-1α pathway may be an effective target. Our experiments provide guidance for future studies on the efficacy of 6G or suitable derivatives in preventing the cardiotoxicity of anticancer drugs.

## Data Availability

The original contributions presented in the study are included in the article/Supplementary Material, further inquiries can be directed to the corresponding authors.
